# Pin1 inhibits PP2A-mediated Rb dephosphorylation in regulation of cell cycle and S-phase DNA damage

**DOI:** 10.1038/cddis.2015.3

**Published:** 2015-02-12

**Authors:** Y Tong, H Ying, R Liu, L Li, J Bergholz, Z-X Xiao

**Affiliations:** 1 Center of Growth, Metabolism and Aging, Key Laboratory of Biological Resources and Ecological Environment of Ministry of Education, College of Life Sciences and State Key Laboratory of Biotherapy, Sichuan University, Chengdu 610064, China; 2 The University of Texas MD Anderson Cancer Center, Houston, TX 77030, USA; 3 Cancer Center, West China Hospital, Sichuan University, Chengdu 610041, China; 4 Department of Biochemistry, Boston University School of Medicine, Boston, MA 02118, USA

## Abstract

Inactivation of the retinoblastoma protein (Rb) has a key role in tumorigenesis. It is well established that Rb function is largely regulated by a dynamic balance of phosphorylation and dephosphorylation. Although much research has been done to understand the mechanisms and function of RB phosphorylation, the regulation of Rb dephosphorylation is still not well understood. In this study, we demonstrate that Pin1 has an important role in the regulation of Rb function in cell cycle progression and S-phase checkpoint upon DNA damage. We show that the Rb C-pocket directly binds to the Pin1 WW domain *in vitro* and *in vivo*, and that the phosphorylation of Rb C-pocket by G1/S Cyclin/Cyclin-dependent kinase complexes is critical for mediating this interaction. We further show that Rb-mediated cell cycle arrest and Rb-induced premature cellular senescence are effectively inhibited by Pin1 expression. In addition, DNA damage induces Rb dephosphorylation in a PP2A-dependent manner, and this process is inhibited by Pin1. Furthermore, the overexpression of Pin1 promotes Rb hyperphosphorylation upon S-phase DNA damage. Importantly, both the Pin1 WW domain and isomerase activity are required for its effect on S-phase checkpoint. Moreover, the overexpression of Pin1 is correlated with Rb hyperphosphorylation in breast cancer biopsies. These results indicate that Pin1 has a critical role in the modulation of Rb function by the regulation of Rb dephosphorylation, which may have an important pathological role in cancer development.

The Retinoblastoma protein (Rb), encoded by the *RB1* gene, is a critical regulator of cell cycle progression and has an important role in numerous aspects of biology, including DNA damage response, apoptosis, senescence and differentiation.^1, 2, 3, 4, 5^ Rb is an important regulator of the cell cycle that acts predominantly by binding to and inhibiting the gene transactivation by E2F transcription factors, which would otherwise induce the expression of genes that enhance cell cycle progression. Rb binds E2F proteins through the Rb large pocket domain (RbLP), which includes the two conserved A and B domains as well as a C-terminal pocket (RbC). The A and B domains, referred together as the Rb small pocket (RbSP), mediate binding to specific regulatory proteins and oncoproteins containing a conserved LXCXE motif.^6, 7, 8, 9^ The Rb C-pocket has been shown to be essential for mediating Rb interaction with E2F.^10^ In addition, the RbC directly binds to MDM2, which inhibits Rb by competing with E2F for binding, as well as by promoting Rb degradation by the proteasome.^11, 12^

The biological function of Rb is critically regulated by protein phosphorylation. Hypophosphorylated Rb interacts with E2F, thereby acting as the biologically active form of Rb. Conversely, hyperphosphorylated Rb is unable to bind E2F proteins, thereby allowing E2F to promote cell cycle progression.^1, 13^ During cell cycle, Rb phosphorylation is primarily conducted by Cyclin/Cyclin-dependent kinase (CDK) complexes;^4, 14, 15, 16^ Cyclin D/CDK4/6 are the initial kinases to phosphorylate Rb, followed by Cyclin E/CDK2 and then by Cyclin A/CDK2. The majority of Cyclin/CDK phosphorylation sites are found in the RbC.^4, 17^ Dephosphorylation of Rb by protein phosphatase 1 (PP1) and protein phosphatase 2A (PP2A) during mitotic exit returns Rb to a hypophosphorylated state, in keeping with the required regulation of a new cell cycle.^18, 19, 20^

Rb has a pivotal role in regulating cell cycle progression during normal and stress conditions. S-phase DNA damage induced by irradiation, oxidative stress or by chemotherapeutic agents such as cisplatin or etoposide, leads to rapid PP2A-dependent Rb dephosphorylation and activation, thus resulting in the suppression of DNA synthesis and cell cycle arrest. Moreover, PP2A has been shown to enhance Rb function toward inhibiting DNA replication via the recruitment of hypophosphorylated Rb to replication control sites.^19, 20, 21, 22^

The prolyl isomerase Pin1 binds to and modulates numerous proteins involved in cell proliferation, differentiation, DNA damage response, apoptosis and development.^23, 24^ Pin1 consists of an N-terminal WW domain for specific protein interaction and a C-terminal catalytic peptidyl-prolyl isomerase (PPIase) domain. Pin1 specifically catalyzes *cis* to *trans* isomerization of proline residues in strictly phosphorylated serine/threonine-proline moieties (pS/T-P), thus affecting substrate function, stability, subcellular localization and/or interacting properties.^25, 26, 27^

In this study, we describe a molecular mechanism by which Pin1 modulates Rb function in cell cycle progression and in DNA damage-induced S-phase checkpoint. We show that Pin1 specifically binds to hyperphosphorylated Rb and inhibits PP2A-mediated Rb dephosphorylation. In addition, Rb-mediated cell cycle arrest and Rb-induced premature cellular senescence are effectively inhibited by Pin1 expression. Similarly, Pin1 deficiency leads to abnormal Rb dephosphorylation upon S-phase DNA damage, resulting in a defective S-phase checkpoint. Hence, this study suggests a novel molecular mechanism in which the Pin1-mediated modulation of Rb phosphorylation has an important role in cancer development.

## Results

### Pin1 specifically binds to the Rb C-pocket

**Figure 1:**
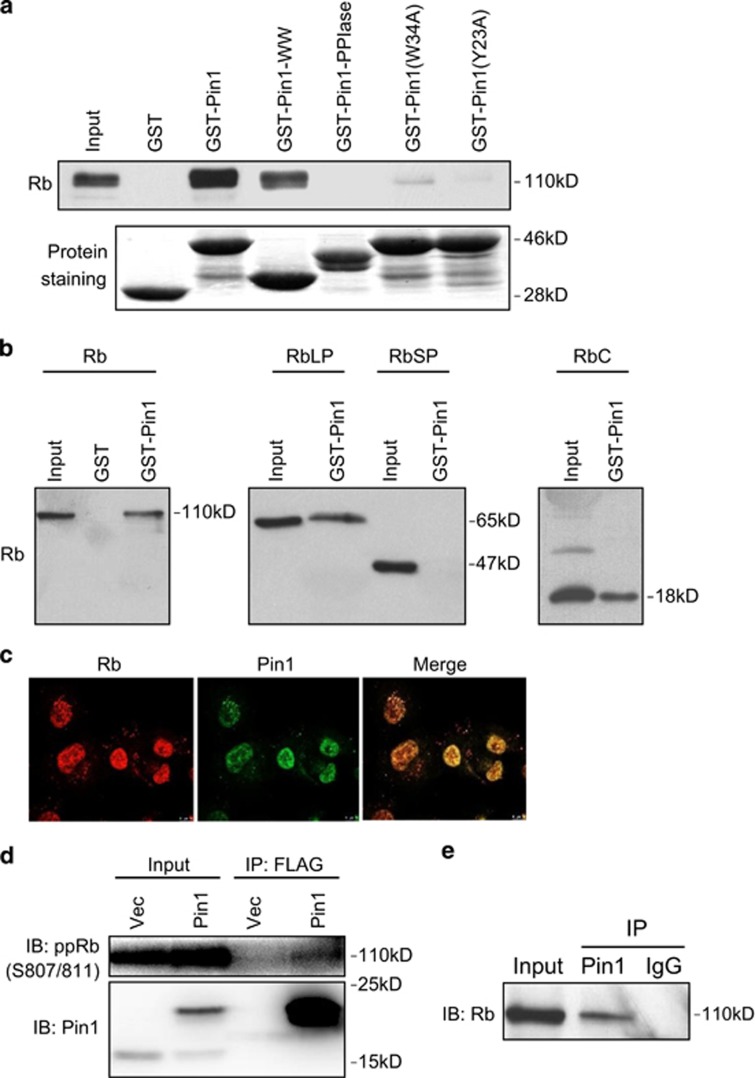
The Pin1 WW domain directly binds to the hyperphosphorylated Rb C-pocket. (**a**) U2-OS cell lysates were incubated with full-length, truncated or mutant Pin1-GST fusion constructs and subsequently subjected to GST pull-down assay, as shown. Proteins were separated by SDS-PAGE and immunoblotted with an Rb-specific antibody (top panel). Comparable levels of input GST or GST fusion proteins are shown by Coomassie blue staining (lower panel). (**b**) U2-OS cells were transiently transfected with full-length Rb, RbLP, Rb small pocket (RbSP) or Rb C-pocket (RbC). Total protein (500 *μ*g) was subjected to GST pull-down assay using recombinant GST-Pin1 or GST as a control and then analyzed by western blotting for Rb binding. Total protein (10 *μ*g) from each cell lysate was directly loaded as input controls. (**c**) H1299 cells were subjected to immunofluorescence, as shown. (d) Cell lysates from H1299 cells stably expressing FLAG-Pin1 or pLVX vector were subjected to immunoprecipitation with anti-FLAG M2 Affinity Gel, and immunoblotted for phosphorylated Rb (pRb) at Ser807/811 (S807/811) or Pin1, as shown. (**e**) U2-OS cell lysates were subjected to immunoprecipitation with a Pin1-specific antibody or a control IgG, and immunoblotted for Rb, as shown

The Rb C terminus contains several S/T-P motifs, which are putative Pin1-binding sites. We therefore examined whether Pin1 can physically interact with Rb using a pull-down assay. As shown in Figure 1a, both GST-Pin1 and GST-Pin1-WW effectively pulled down endogenous Rb from osteosarcoma U2-OS cell lysates, whereas GST-Pin1-PPIase domain was unable to bind Rb. In addition, point mutations in the Pin1 WW domain at W34A or Y23A, two amino-acid residues critical for Pin1 substrate binding,^28^ abolished Pin1–Rb interaction (Figure 1a). These results indicate that Rb interacts specifically with the Pin1 WW domain.

To further define the Pin1–Rb interaction, we expressed various Rb protein constructs in U2-OS cells and subjected the cell lysates to GST-Pin1 pull down. As shown in Figure 1b, Pin1 interacted with full-length Rb as well as with the RbLP (including A, B and C domains), but not with the Rb small pocket (RbSP, including A and B domains), suggesting that the Rb C-pocket (RbC) is important for Pin1 interaction. Indeed, the RbC alone interacted well with Pin1, indicating that the Rb C-pocket is necessary and sufficient for Rb-Pin1 interaction. Moreover, we observed endogenous Pin1–Rb co-localization by immunofluorescence in non-small-cell lung carcinoma H1299 cells (Figure 1c), as well as binding *in vivo* by co-immunoprecipitation in H1299 and U2-OS cells (Figures 1d and e).

### Pin1–Rb interaction is induced by G1-S Cyclin-mediated Rb phosphorylation

**Figure 2:**
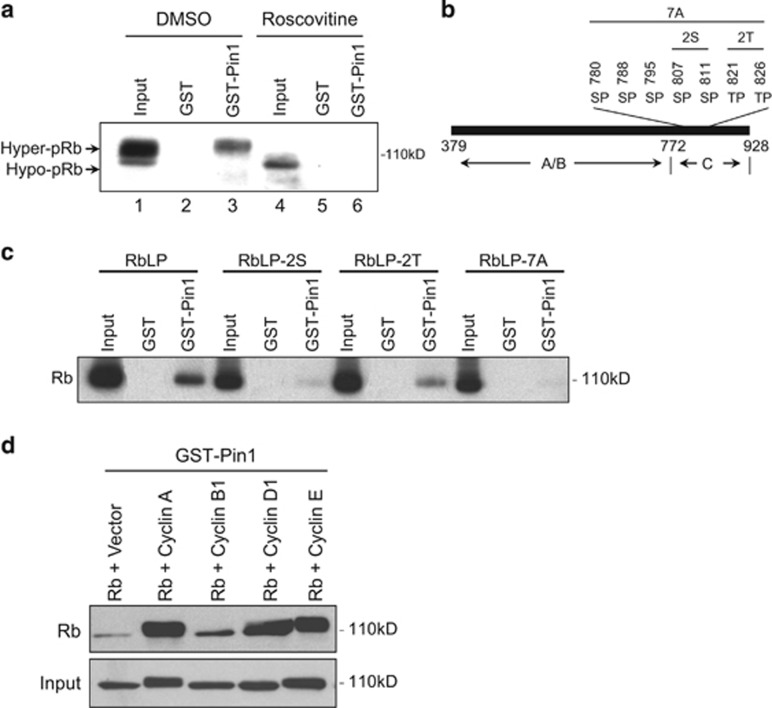
Pin1–Rb interaction is induced by G1-S Cyclin-mediated phosphorylation. (**a**) U2-OS cells were treated with vehicle (dimethyl sulfoxide, DMSO) or 30 *μ*M roscovitine for 18 h. Cell lysates (500 *μ*g total protein) were pulled down by GST-Pin1, followed by western blotting for Rb. Total protein (30 *μ*g) was loaded as an input control. Hyper-pRb, hyperphosphorylated Rb; Hypo-pRb, hypophosphorylated Rb. (**b**) Diagram of RbLP highlighting seven pS/T-P putative Pin1-binding sites within the Rb C-pocket (RbC), as well as two double-point mutations to Ala (S807A;S811A, denominated 2S, and T821A;T826A, denominated 2T) and mutation of all Ser or Thr residues to Ala residues (7A) used for RbC-Pin1-binding studies. (**c**) U2-OS cells were transfected with WT or mutant RbLP. Cell lysates were subjected to GST-Pin1 pull-down assay and western blotted for Rb to assess binding. (**d**) Saos-2 cells were co-transfected with Rb along with Cyclin A, Cyclin B1, Cyclin D1, Cyclin E or a vector control, as shown. Cell lysates were subjected to GST-Pin1 pull down followed by western blotting for Rb

CDKs are primarily responsible for the phosphorylation of Rb. As the majority of CDK phosphorylation sites are within the Rb C-pocket,^4, 17^ we examined whether CDK-mediated Rb phosphorylation signals for Pin1–Rb interaction. Pin1 preferentially bound to the hyperphosphorylated, slower migrating Rb species. Treatment of U2-OS cells with roscovitine, a potent CDK inhibitor, resulted in significant accumulation of hypophosphorylated Rb, as shown by the faster migration of hypophosphorylated Rb species. Roscovitine treatment abrogated Pin1–Rb interaction, indicating that Rb phosphorylation is required for Pin1–Rb binding, and that Pin1 preferentially binds to hyperphosphorylated Rb but not to hypophosphorylated Rb (Figure 2a).

The Rb C-pocket contains seven pS/T-P motifs (Figure 2b). To investigate the role of these putative Pin1-binding sites in Pin1–Rb interaction, we expressed wild-type (WT) or mutant RbLP in U2-OS cells and compared their ability to bind Pin1. Double substitution of S807 and S811 by alanine residues (RbLP-2S) virtually abolished Pin1–Rb interaction. Likewise, combined T821A and T826A mutations (RbLP-2T) significantly inhibited Pin1–Rb binding, albeit to a lesser degree. Moreover, RbLP-7A, in which all seven serine or threonine residues were substituted by alanine, completely lost interaction with Pin1 (Figure 2c).

Next we examined the effect of specific Cyclin/CDK complexes on Rb-Pin1 interaction. We co-transfected Saos-2 cells with Rb and various Cyclin-expressing plasmids, and subjected the cell lysates to GST-Pin1 pull-down assays followed by immunoblotting for Rb. We chose to use Saos-2 cells because they are Rb-null and exhibit low levels of endogenous CDK activity. As shown in Figure 2d, the co-expression of Rb with Cyclin D1, Cyclin E or Cyclin A dramatically increased Pin1–Rb interaction, whereas the co-expression of Cyclin B1 had little effect, despite comparable levels of protein expression. These data indicate that the phosphorylation of Rb by G1- and S-phase Cyclin-associated kinases is a crucial signal for Rb-Pin1 interaction.

### Pin1 inhibits PP2A-mediated Rb dephosphorylation

**Figure 3:**
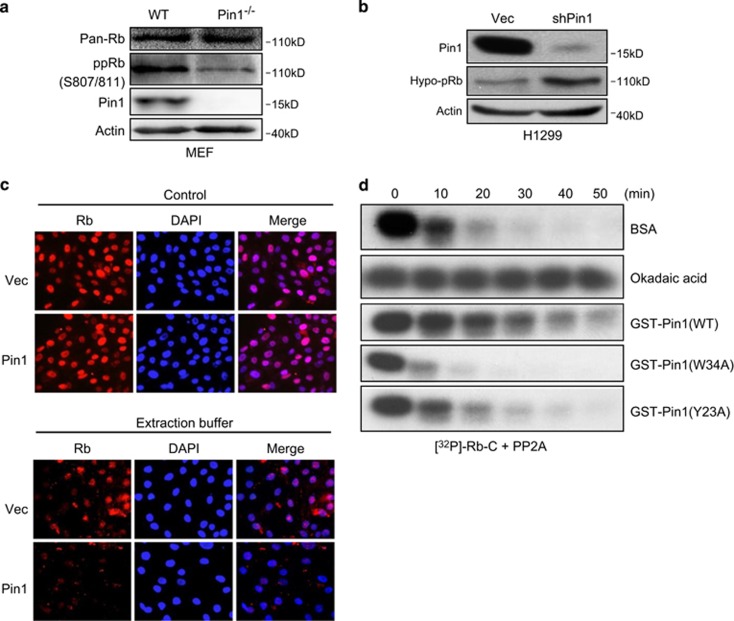
Pin1 inhibits Rb PP2a-mediated dephosphorylation. (**a**) WT or Pin1-deficient (Pin1^−/−^) MEF cell lysates were subjected to western blot analysis for total Rb levels (Pan-Rb), phosphorylated Rb (pRb) at Ser807/811 (S807/811), Pin1 or Actin. (**b**) H1299 cells were infected with recombinant retrovirus expressing shRNA against Pin1 or a vector control. Whole-cell lysates were subjected to western blotting, as shown. (**c**) MCF-10A cells were stably transfected with a PLVXpuro vector control (Vec) or PLVX-Pin1 (Pin1). Stable cells were treated with or without extraction buffer and subsequently subjected to immunofluorescence for Rb and counterstained with DAPI. (**d**) Rb C-pocket (RbC) peptides were *in vitro* phosphorylated and labeled with Cyclin E/CDK2 complexes with *γ*-[^32^P]-ATP as described in the Materials and Methods. [^32^P]-labeled phosphorylated RbC peptides were then incubated with BSA, okadeic acid or WT or mutant GST-Pin1 fusion proteins, before incubation with PP2A for the indicated times. Dephosphorylation reactions were quenched by the addition of SDS sample buffer. Samples were analyzed by SDS-PAGE followed by autoradiography

As our results showed that Pin1 binds to hyperphosphorylated Rb, we investigated whether the Pin1–Rb interaction affects Rb phosphorylation. As shown in Figure 3a, Pin1-deficient mouse embryonic fibroblast (MEF) cells exhibited a clear decrease in the hyperphosphorylated Rb species at Ser807 and Ser811. Furthermore, Pin1 ablation resulted in increased hypophosphorylated Rb levels in H1299 cells (Figure 3b). Hypophosphorylated Rb associates tightly with the nuclear envelope and requires high salt concentrations for dissociation, whereas hyperphosphorylated Rb is soluble in lower salt concentrations.^29^ Thus, to further examine the effect of Pin1 on Rb phosphorylation, we treated human nontransformed mammary epithelial MCF-10A cells expressing Pin1 or a vector control with or without extraction buffer containing 0.1% Triton-X-100 and 250 mM NaCl. Treatment of Pin1-expressing cells with extraction buffer resulted in Rb dissociation from the nucleus to a much greater extent than in control cells (Figure 3c), indicating that Rb binds less tightly to the nuclear matrix upon Pin1 expression. Together these data indicate that Pin1 is important in maintaining hyperphosphorylation of Rb.

As it has been shown that Pin1 regulates PP2A activity,^26, 30, 31^ and that PP2A is important for Rb dephosphorylation,^19, 20^ we performed *in vitro* dephosphorylation experiments to examine the effect of Pin1 on PP2A-mediated Rb dephosphorylation. RbC fragments were *in vitro* phosphorylated and radiolabeled using [^32^P] by Cyclin E/CDK2, preincubated with WT or mutant Pin1, and incubated with PP2A for the indicated time intervals, followed by subsequent SDS-polyacrylamide gel electrophoresis (SDS-PAGE) and autoradiography. Okadaic acid, which specifically inhibits PP2A activity at low concentrations,^32^ or bovine serum albumin (BSA) were used in parallel as controls. As shown in Figure 3d, [^32^P]-labeled RbC was rapidly dephosphorylated by PP2A when preincubated with BSA, whereas preincubation with okadaic acid completely blocked Rb dephosphorylation. Strikingly, dephosphorylation of Rb by PP2A was significantly inhibited by WT Pin1, but not by the mutant derivatives. These results indicate that Pin1 is capable of inhibiting PP2A-mediated Rb dephosphorylation *in vitro*.

### Pin1 inhibits Rb-mediated cell cycle arrest and Rb-induced senescence

**Figure 4:**
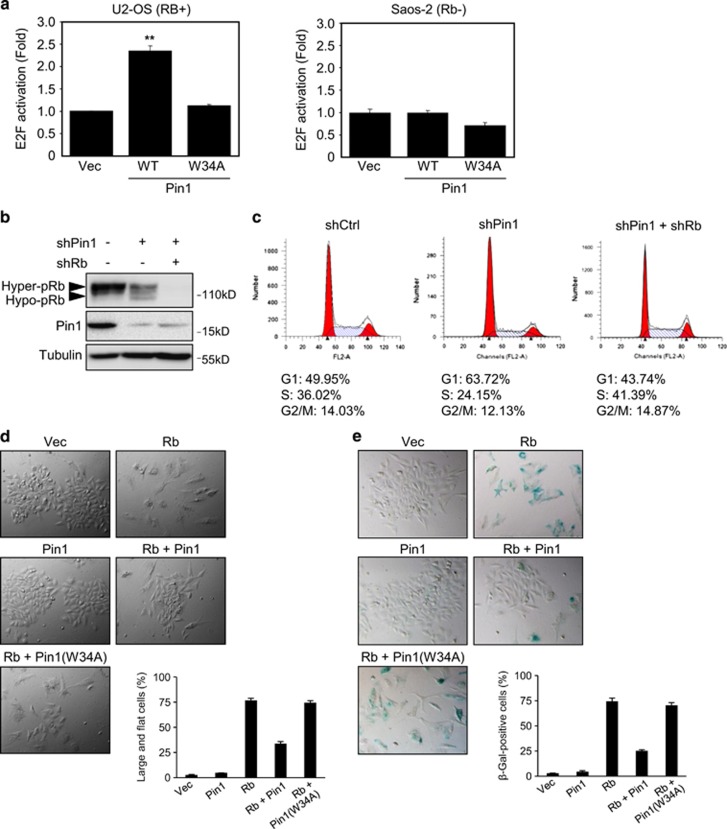
Pin1 inhibits Rb function in cell cycle progression and senescence. (**a**) U2-OS (Rb-positive) or Saos-2 (Rb-null) cells were co-transfected with WT or mutant Pin1, or a vector control, along with an E2F1-responsive DHFR-Luciferase (DHFR-Luc) reporter and a *β*-Galactosidase expression plasmid. Cells were lysed 24 h after transfection and subjected to luciferase and *β*-Galactosidase activity assays. DHFR-Luc activity was normalized to *β*-Galactosidase activity as described in the Materials and Methods and presented as fold activation. Results presented as means and S.E. of three independent experiments performed in triplicate. **P<0.01. (**b**) H1299 cells were stably infected with shRNA against Rb, Pin1 or a control, and selected by puromycin resistance. Cell lysates were subjected to western blotting, as indicated. (**c**) Stable H1299 cells were subjected to flow cytometry for analysis of cell cycle stage. (**d** and **e**) Saos-2 cells were co-transfected with Rb and/or WT or mutant Pin1, or with a vector control, and selected by puromycin resistance, as described in the Materials and Methods. Stable cells were grown in normal growth media supplemented with 0.5 *μ*g/ml puromycin for 10 days. Cells were then visualized under a light microscope for the analysis of large/flat phenotype (**d**) or were subjected to *β*-Galactosidase staining (**e**). Cells were scored and presented as the percentage of stained cells over total cells. Results are presented as representative images and mean and S.E. from three independent experiments. At least 300 cells were counted for each condition

As our data show that Pin1 inhibits Rb dephosphorylation, thereby maintaining Rb hyperphosphorylated, which is unable to bind E2F, we investigated the effect of Pin1 on Rb function toward inhibiting E2F activity. To this end, we used a luciferase reporter (DHFR-Luc) driven by the promoter of dihydrofolate reductase, which is a bona fide downstream target of E2F. We co-transfected DHFR-Luc with either WT Pin1 or Pin1-W34A into Rb-positive U2-OS cells, followed by luciferase activity assays. WT Pin1, but not Pin1-W34A, significantly stimulated E2F reporter activity in U2-OS cells. On the other hand, the ectopic expression of Pin1 in Rb-null Saos-2 cells failed to induce E2F reporter activity (Figure 4a). To assess the biological significance of this regulation, we silenced Rb and/or Pin1 expression in H1299 cells. Again, Pin1 ablation led to increased hypo-pRb and decreased hyper-pRb levels (Figure 4b). Flow cytometry analysis of these cells showed that Pin1 knockdown resulted in G1 cell cycle arrest, which was effectively reverted upon concomitant Rb knockdown (Figure 4c). Moreover, the ectopic Rb expression in Saos-2 cells (Rb-null and p53 null) has been shown to induce premature cellular senescence.^33^ In keeping with these observations, Rb expression in Saos-2 cells led to an increased cellular senescence, as manifested by flat and enlarged cell morphology (Figure 4d), and increased senescence-associated *β*-Galactosidase (SA-*β*-Gal) activity (Figure 4e). Notably, concomitant Pin1 expression in Rb-expressing cells significantly reversed these effects, whereas mutant Pin1(W34A) was unable to affect Rb-induced cellular senescence (Figures 4d and e). These data suggest that Pin1 inhibits Rb-mediated cell cycle arrest and Rb-induced senescence.

### Pin1 modulates Rb phosphorylation during cell cycle progression

**Figure 5:**
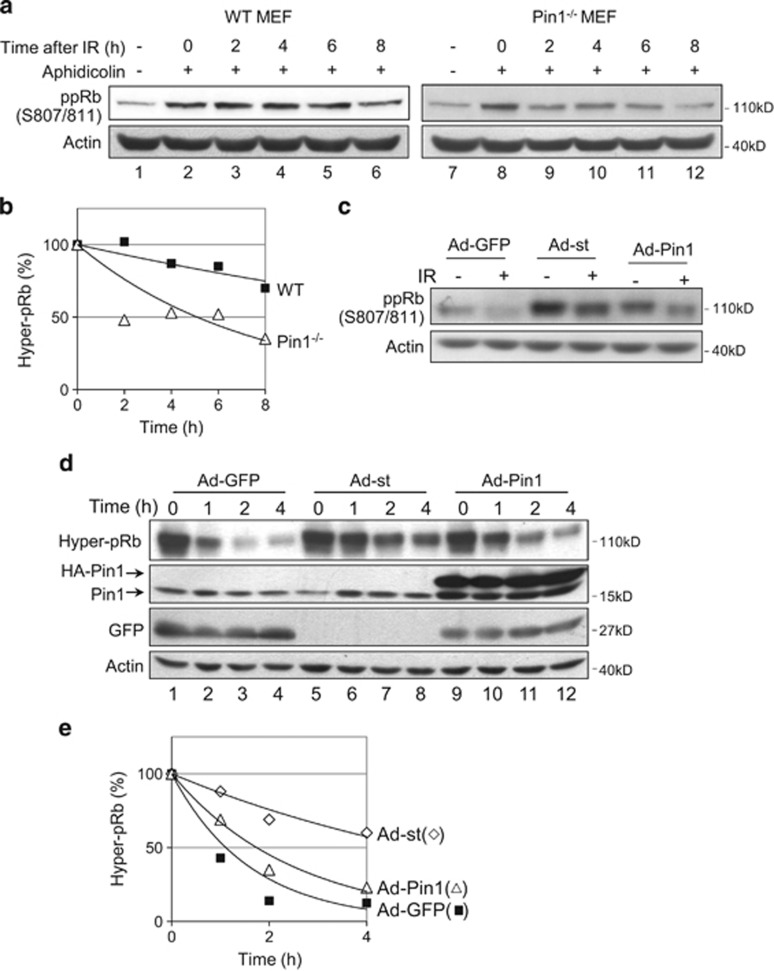
Pin1 modulates Rb dephosphorylation in cell cycle control. (**a**) WT or Pin1^−/−^ MEF cells were synchronized with aphidicolin and then *γ-*irradiated (20 Gy). Samples were collected every 2 h for 8 h and subjected to western blotting, as shown. (**b**) Rb phosphorylation was quantitated by densitometry analysis and expressed as a percentage of Rb phosphorylation at time zero. (**c**) IMR90 cells were stably infected with adenovirus expressing GFP and HA-tagged Pin1 (Ad-Pin1), SV40 small t antigen alone (Ad-st), or GFP alone (Ad-GFP). Cells were synchronized with 1.0 mM hydroxyurea for 18 h, released and then *γ*-irradiated (20 Gy). Samples were collected 6 h post irradiation and subjected to western blotting, as shown. (**d**) H1299 cells were stably infected with Ad-GFP, Ad-st or Ad-Pin1. Six hours post infection, cells were synchronized with 100 ng/ml nocodazole for 18 h. Mitotic cells were shaken off and grown in fresh media for the indicated times. Whole-cell lysates were subjected to western blotting as indicated. Rb phosphorylation was quantitated by densitometry analysis and expressed as a percentage of Rb phosphorylation at time zero. (**e**) Rb phosphorylation was quantitated by densitometry analysis, normalized to actin and expressed as a percentage of Rb phosphorylation at time zero

Rb has an important role at the S-phase checkpoint in cellular response to DNA damage. Upon *γ*-irradiation or oxidative stress, Rb is activated via dephosphorylation, resulting in blockage of DNA synthesis and S-phase cell cycle arrest.^19, 20^ As Pin1 modulates Rb phosphorylation, we investigated the role of Pin1 in the regulation of Rb phosphorylation during S-phase checkpoint control. WT or Pin1^−/−^ MEF cells were synchronized at S-phase by treatment with aphidicolin, which led to increased hyperphosphorylated Rb in both WT and Pin1^−/−^ MEFs (Figure 5a, lanes 2 and 8), indicating that Rb was hyperphosphorylated during S-phase. *γ*-Irradiation of S-phase WT MEFs resulted in Rb dephosphorylation at 8 h post irradiation. In contrast, Rb dephosphorylation in Pin1^−/−^ MEFs was evident as early as 2 h post irradiation, yielding markedly decreased Rb phosphorylation levels in comparison with WT MEFs (Figures 5a and b).

We examined whether the ectopic expression of Pin1 can affect Rb phosphorylation at S-phase checkpoint. IMR90 cells stably expressing either Pin1 or GFP were synchronized by hydroxyurea (HU) and then released into S-phase before exposure to *γ*-irradiation. As a control, we analyzed the cells expressing the SV40 small t antigen (st), which has been shown to inhibit PP2A activity, thus leading to higher levels of hyperphosphorylated Rb.^34, 35^ *γ*-Irradiation dramatically reduced hyperphosphorylated Rb. Expression of SV40 st led to significantly increased hyperphosphorylated Rb levels upon *γ*-irradiation. Similarly, ectopic expression of Pin1 resulted in Rb hyperphosphorylation in S-phase, albeit less than in cells expressing SV40 st (Figure 5c). Taken together, these data suggest that Pin1, like SV40 st, can sustain Rb phosphorylation via the inhibition of PP2A in response to S-phase DNA damage.

As it has been shown that Rb remains hyperphosphorylated throughout the cell cycle until late M-phase,^15, 16^ we investigated whether Pin1 affected Rb dephosphorylation at M-phase. H1299 cells stably expressing GFP, st or Pin1 were synchronized with nocodazole and then released. As expected, Rb was rapidly dephosphorylated upon release from nocodazole in control cells (Figures 5d and e), whereas SV40 st dramatically inhibited dephosphorylation. Similarly, Pin1 expression delayed Rb dephosphorylation (Figures 5d and e). Thus, these data suggest that Pin1 has a role in the regulation of Rb phosphorylation at mitotic exit.

### Pin1 has a role at S-phase checkpoint

**Figure 6:**
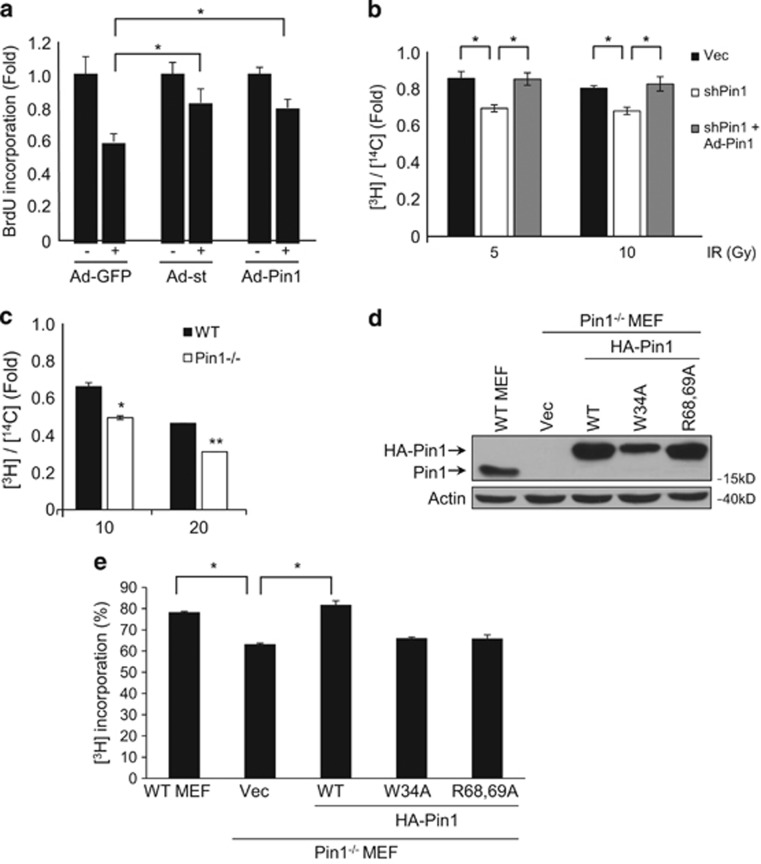
Pin1 has a critical role in S-phase checkpoint control. (**a**) H1299 cells were infected with adenovirus expressing GFP and HA-tagged Pin1 (Ad-Pin1), SV40 small t antigen alone (Ad-st) or GFP alone (Ad-GFP). Cells were synchronized with 1.0 mM HU for 18 h and then released, followed by *γ*-irradiation and labeled with 5-bromo-2'-deoxyuridine (BrdU). Cells were then fixed, stained with anti-BrdU antibody, counterstained with DAPI and visualized by fluorescent microscopy. Results were normalized to untreated controls. At least 100 cells were scored for each condition. Results are expressed as means and S.E. from three independent experiments. *P<0.05. (**b**) H1299 cells were stably transfected with shRNA against Pin1 (shPin1) or a vector control (Vec), and with adenovirus encoding Pin1 (Ad-Pin1), as indicated. Stable cells were subjected to RDS assay as following: cells were prelabeled with [^14^C]-thymidine to provide an internal control for DNA content, synchronized with 1.0 mM HU for 18 h and then released. S-phase cells were exposed to graded doses of *γ*-irradiation and then pulse labeled with [^3^H]-thymidine for 15 min. Relative DNA synthesis was determined as the ratio of [^3^H]-thymidine to [^14^C]-thymidine incorporation and normalized to the corresponding nonirradiated control. (**c**) RDS assays were performed in WT or Pin1^−/−^ MEF cells as described above. *P<0.05; **P<0.01. (**d** and **e**) WT or Pin1^−/−^ MEF cells were transfected with HA-tagged WT or mutant Pin1 (W34A or R68,69A), or with a vector control, as shown. (**d**) Protein expression was assessed by western blotting. (**e**) Cells were subjected to RDS assays as described above. Results presented as mean [^3^H]-thymidine incorporation and S.E. from three independent experiments

As Rb has a critical role in S-phase checkpoint control upon DNA damage, and our data indicate that Pin1 can regulate Rb activity via modulation of Rb phosphorylation, we investigated the role of Pin1 in checkpoint control upon DNA damage during S-phase. H1299 cells stably expressing Pin1, SV40 st or GFP were synchronized at S-phase with HU, and subsequently released and then exposed to 20 Gy *γ*-irradiation. Cells were then labeled with BrdU. *γ*-Irradiation led to marked inhibition of DNA synthesis in control cells, as expected, whereas the expression of either SV40 st or Pin1 resulted in higher levels of BrdU incorporation upon DNA damage (Figure 6a), suggesting that Pin1 can impede S-phase checkpoint in response to DNA damage.

Next we examined the effects of silencing Pin1 in IR-induced S-phase checkpoint using radioresistant DNA synthesis (RDS) assays. Stable cells expressing shRNA specific for Pin1 were first labeled with methyl-[^14^C]-thymidine, synchronized at S-phase and released, and then subjected to *γ*-irradiation (5 or 10 Gy), followed by pulse labeling with methyl-[^3^H]-thymidine to determine the amount of ongoing DNA synthesis after DNA damage. Exposure to 5 and 10 Gy *γ*-irradiation led to ~15 and 20% reductions in [^3^H]-thymidine incorporation, respectively. Notably, Pin1 ablation further reduced DNA synthesis upon DNA damage, resulting in over 30% reductions in [^3^H]-thymidine incorporation. In addition, these effects were effectively reverted by reconstituting Pin1 expression (Figure 6b). These data indicate that Pin1 has an important role in IR-induced S-phase checkpoint.

To further examine the role of Pin1 in S-phase checkpoint control, we conducted RDS assays in Pin1-deficient and WT MEFs. Although *γ*-irradiation of WT MEF cells led to a dose-dependent decrease in DNA synthesis, Pin1 deficiency led to further reduced DNA synthesis (Figure 6c). Next we reintroduced either WT Pin1, Rb binding-deficient Pin1(W34A), or Pin1(R68,69A) defective in isomerase activity,^36^ into Pin1^−/−^ MEFs. Expression of WT and mutant Pin1 was confirmed by western blotting (Figure 6d). Consistently, RDS assays showed that Pin1 deficiency led to reduced [^3^H]-thymidine incorporation upon *γ*-irradiation, which was reverted by WT Pin1, but not by Pin1(W34A) or Pin1(R68/69A) (Figure 6e). These data suggest that both the ability of Pin1 to bind Rb and its isomerization activity are essential for Pin1 function on S-phase checkpoint control.

### Pin1 overexpression correlates with hyperphosphorylation of Rb in human breast cancer

**Figure 7:**
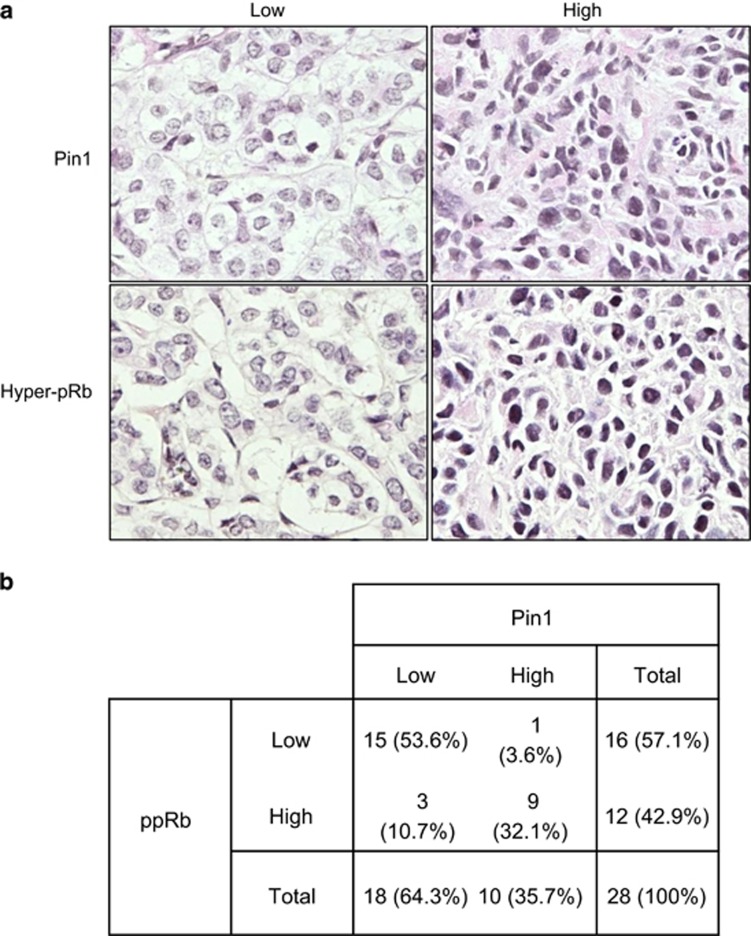
Pin1 overexpression correlates with the hyperphosphorylation of Rb in human breast cancer. (**a**) Consecutive breast cancer sections were subjected to immunohistochemistry for Pin1 expression or hyperphosphorylated Rb (pS807/811) levels, and photographed under a light microscope. Micrographs were then analyzed and classified as high or low, depending on intensity of staining and percentage of positive cells. Representative images for each level are shown. (**b**) Summary of Pin1 expression and hyperphosphorylated Rb (pS807/811; ppRb) levels. Correlation was analyzed by the Spearman rank test (*P*<0.001)

Pin1 is frequently overexpressed in a variety of human cancers, including breast cancers.^37^ As our results show that Pin1 inhibits Rb function by maintaining Rb hyperphosphorylation, we examined whether there is a clinical relevance. We performed immunohistochemical staining of human breast tumor biopsy samples and scored for Pin1 and Rb phosphorylation (pS807/811). As shown in Figure 7a, hyperphosphorylation of Rb on S807/S811 was found in 42.9% of all samples (12 out of 28), whereas the overexpression of Pin1 was found in ~35% of all samples (10 out of 28). Notably, almost all tumors containing higher levels of Pin1 (9 out of 10) exhibited markedly higher levels of hyperphosphorylated Rb (pS807/811), whereas tumors that expressed lower levels of Pin1 (15 out of 18) similarly exhibited lower levels of hyperphosphorylated Rb. Statistical analysis showed a significant positive correlation between Pin1 expression and Rb hyperphosphorylation, as determined by a Spearman rank correlation test (*P*<0.001; Figure 7b and Supplementary Figure S1). Taken together, these results indicate that the overexpression of Pin1 is concomitant with Rb hyperphosphorylation in human breast cancer.

## Discussion

The Rb protein is critically important for regulating cell cycle progression and DNA damage response. Hence, the tight regulation of Rb activity is crucial for preventing cellular transformation and tumorigenesis. We demonstrated here that Pin1 has an important role in Rb-mediated cell cycle regulation and S-phase checkpoint upon DNA damage. First, Pin1 specifically binds to the RbC and this interaction is dependent on Rb phosphorylation induced by G1/S cyclins. Second, Pin1 inhibits PP2A-mediated Rb dephosphorylation *in vitro*. Third, Pin1 expression effectively inhibits Rb-induced premature cellular senescence. Fourth, the overexpression of Pin1 promotes Rb hyperphosphorylation and leads to defective S-phase check point upon DNA damage. Furthermore, Pin1 overexpression significantly correlates with Rb hyperphosphorylation in human breast cancer biopsy samples.

Multiple proteins have been shown to interact with hypophosphorylated Rb, which is the biologically active species of Rb. To date, except for protein phosphatases, there are no known cellular proteins that specifically interact with hyperphosphorylated Rb. Here we demonstrated that Pin1 specifically binds to RbC in a phosphorylation-dependent manner. Notably, the RbC contains multiple S/T-P moieties that are phosphorylated by Cyclin/CDK complexes and have a critical role in regulating Rb function.^17, 38^ We found that mutation of these sites to alanine abolished Pin1–Rb interaction, whereas the expression of Cyclin A, Cyclin D1 or Cyclin E enhances Pin1–Rb binding. These observations are consistent with a recent report showing that CDK2 or CDK4/6 expression strengthens Rb-Pin1 interaction in glioblastoma multiforme cell lines.^39^ Of note, the authors also reported that the Rb small pocket, which excludes the RbC, interacts with Pin1, as shown by GST pull-down assays in 293FT cells.^39^ The reasons for these discrepancies are unknown. Although these studies demonstrate that CDK-mediated Rb hyperphosphorylation is a prerequisite for Pin1–Rb interaction, the molecular mechanisms responsible for Pin1-mediated regulation of Rb function have not yet been elucidated. Rb function is determined by its phosphorylation status, which depends on the kinetics of phosphorylation by Cyclin/CDK complexes and dephosphorylation by phosphatases, such as PP1 and PP2A. Here we found that WT Pin1, but not Pin1 mutant derivatives defective in Rb binding, inhibit Rb function in cell cycle progression and in premature cellular senescence. Moreover, Pin1 prevents Rb dephosphorylation at S-phase checkpoint, thus preventing Rb-mediated cell cycle arrest. Our data indicate that Pin1 directly inhibits PP2A, therefore maintaining Rb hyperphosphorylation. Interestingly, PP2A normally dephosphorylates phospho-Ser/Thr-Pro isomers in the *trans* conformation,^26, 40^ and Pin1-mediated *cis*–*trans* proline isomerization generally promotes PP2A-mediated dephosphorylation of a number of proteins, such as Pim-1 kinase,^41^ raf-1,^30^ c-Myc,^31^ Cdc25C^26^ and Tau.^26^ Nevertheless, Pin1 has also been shown to inhibit PP2A-mediated dephosphorylation of neurofilament proteins in cortical neurons.^42^ Hence, the specific molecular mechanisms underlying inhibition of PP2A by Pin1 require further investigation. It is possible that specific conformational changes upon Pin1-mediated isomerization might mask docking sites for PP2A and, therefore, the outcome of Pin1 action for PP2A may vary depending on the substrates and signaling context.

Nonetheless, Pin1 may enhance Rb hyperphosphorylation by other mechanisms. For example, Pin1 has been reported to induce Cyclin D1 expression and enhance its function.^27, 37, 43^ Thus, it is possible that Pin1 modulates Rb phosphorylation by combined effects of Cyclin D1 activation and PP2A inhibition. However, Cyclin D proteins have a major role in mediating Rb phosphorylation during G1 phase, but less likely during S-phase. Interestingly, it was reported that S-phase DNA damage downregulates CDK2 and Cyclin D1.^44, 45^ Thus, it could be that reduction of Rb phosphorylation upon S-phase DNA damage is a consequence of reduced Cyclin/CDK activity. However, DNA damage-induced accumulation of hypophosphorylated Rb during S-phase does not require CDK inhibitors p16^INK4A^ or p21^WAF1/CIP1^ (ref. 21). Therefore, although a potential contribution by changes in CDK activity cannot be completely ruled out, it is likely that the observed Rb hypophosphorylation upon S-phase DNA damage results mainly from PP2A-induced dephosphorylation and that Pin1 inhibits this process by directly interacting with hyperphosphorylated Rb.

In addition to having a role in S-phase DNA damage control, we showed that Pin1 inhibits Rb dephosphorylation at mitotic exit. It is conceivable that Pin1 is involved in temporal regulation of Rb dephosphorylation, and that abnormally high Pin1 levels may disrupt this process, leading to defects in cell cycle progression.

Rb dysregulation is directly linked to tumorigenesis and cancer progression. Germline mutations in the *RB1* gene often result in childhood retinoblastoma.^46^ Similarly, *RB1* mutations have also been found in numerous human cancers, including small-cell lung carcinoma, osteosarcoma and melanoma.^47, 48, 49^ Furthermore, aberrations leading to the abnormal activation of Rb kinases, such as cyclin D1 overexpression and inactivation of p16^INK4A^, are frequently found in human cancers.^50^ Similarly, Pin1 overexpression is often correlated with cancer progression.^37, 51^ Moreover, Pin1 was shown to enhance tumorigenesis and metastasis induced by mutant p53 in mouse models.^52^ However, much less is known about the link between Rb dephosphorylation and tumorigenesis. Here we found that the overexpression of Pin1 correlates with increased Rb phosphorylation in breast cancer. We previously reported that Pin1 is overexpressed and closely correlated with Rb phosphorylation in DMBA-induced tumors in mice, compared with normal mammary glands.^53^ Also, Pin1 expression correlates with Rb phosphorylation in human glioma samples,^54^ suggesting that increased Pin1 expression could account, at least in part, for Rb inactivation. Notably, analyses of TCGA database^55^ reveals a clear correlation between Pin1 mRNA levels and phosphoralated Rb (S807/811) in Glioblastoma (*N*=70, *P*<0.01). However, analysis of the TCGA database does not reveal a clear correlation in invasive breast adenocarcinoma^56^. The reasons for the discrepancy between this analysis and this study are not yet clear. It is possible that Pin1 mRNA levels do not reflect actual protein levels, as reported before^57^. Together this study suggests that Pin1 overexpression and subsequent sustaining of Rb hyperphosphorylation may have an important pathological role in cancer development.

## Materials and Methods

### Cell culture and synchronization

Human osteosarcoma Saos-2 and U2-OS cells and human non-small-cell lung carcinoma H1299 cells were maintained in Dulbecco's modified Eagle medium (DMEM; Invitrogen Inc., Carlsbad, CA, USA) containing 4.5 g/l glucose supplemented with either 10% newborn calf serum (NCS; U2-OS) or 10% fetal bovine serum (FBS; Saos-2) or 5% FBS (H1299). Human nontransformed mammary epithelial MCF-10A cells were maintained in 1 : 1 mixture of DMEM and Ham,s F12 medium with reduced Ca^2+^ (0.04 mM; Invitrogen Inc.), 20 ng/ml epidermal growth factor (Invitrogen Inc.), 100 ng/ml cholera toxin (Sigma, St. Louis, MO, USA), 10 *μ*g/ml insulin (Sigma), 500 ng/ml (95%) hydrocortisone (Sigma) and 5% of Chelex-treated horse serum (Invitrogen Inc.). Primary human lung fibroblast IMR90 cells were maintained in DMEM supplemented with 10% FBS and 0.1 mM nonessential amino acids (GIBCO Life Technologies, Rockville, MD, USA). WT and Pin1-null MEFs were maintained in DMEM supplemented with 10% FBS. For synchronization studies, cells were grown to 60% confluence and treated with 1.0 mM HU for 18 h or 2.0 *μ*g/ml aphidicolin for 24 h or 100 ng/ml nocodazole for 18 h. Cells were released by washing twice with phosphate-buffered saline (PBS) and once with growth media, and then incubated in normal growth media.

### Plasmid transfections, viral infection and RNA interference

Transient transfections were carried out using FuGENE 6 (Roche, Indianapolis, IN, USA) for U2-OS cells, Lipofectamine 2000 (Invitrogen Inc.) for H1299, MEF and Saos-2 cells (where indicated) or CalPhos mammalian transfection kit (Clontech, Mountain View, CA, USA) for Saos-2 cells, according to manufacturer's instructions. Transient expression plasmids include pCMV-Rb (full length, and deletion or point mutant), HA-tagged pHook2-Pin1 (WT and point mutant), PLVXpuro-Pin1 (WT or point mutant) and adenoviral expression plasmids include Pin1-pAdTrack-CMV, SV40 small t antigen (st)-pAdTrack-CMV, and GFP-pAdTrack-CMV. These plasmids were described previously.^11, 25^ Short hairpin RNA against Pin1 (5'-AGGCCGAGTGTACTACTTCAA-3') was cloned into a pSM2 retroviral vector (Thermo Fisher Scientific, Waltham, MA, USA). Retrovirus and adenovirus were amplified by transfecting 293FT cells with the corresponding backbone, and packaging plasmids; supernatants were collected after 48 h, filtered, and either used immediately or stored at −80 °C. Retrovirus were concentrated by ultracentrifugation at 27 000 r.p.m. for 90 min at 4 °C. For viral transductions, cells at 60% confluency were incubated with retrovirus (viral titers of 10^8^ to 10^9^ CFU/ml) or adenovirus (MOI of 1 : 100) for 90 min at 37 °C with mild agitation at 20-min intervals, followed by the addition of culture media supplemented with 10 *μ*g/ml polybrene and incubation for 24 h.

### Luciferase activity assays

U2-OS or Saos-2 cells in six-well plates were co-transfected with 1.5 *μ*g (U2-OS) or 4.0 *μ*g (Saos-2) WT or mutant HA-tagged pHook2-Pin1, along with 0.5 *μ*g (U2-OS) or 2.0 *μ*g (Saos-2) DHFR-Luciferase, and 100 ng (U2-OS) or 400 ng (Saos-2) pCMV-*β*-Galactosidase plasmids, and harvested 24 h after transfection. Luciferase activity was measured using a Luciferase Assay System kit (Promega, Madison, WI, USA) according to the manufacturer's instructions, and normalized to *β*-Galactosidase activity. For *β*-Galactosidase activity, 30 *μ*l cell lysates were incubated in 500 *μ*l LacZ buffer (60 mM Na_2_PO_4_, 40 mM NaH_2_PO_4_, 10 mM KCl and 1.0 mM MgSO_4_, pH 6.95) and 100 *μ*l substrate solution (2 mg/ml *O*-Nitrophenyl *β*-D-Galactopyranoside in 60 mM Na_2_HPO_4_ and 40 mM NaH_2_PO_4_) at 37°C for 20 min. The reaction was stopped by adding 250 *μ*l 1.0 M Na_2_CO_3_ and absorbance was measured at 420 nm.

### Western blot analyses, immunoprecipitation and immunofluorescence experiments

Unless indicated, cells were lysed in EBC_250_ lysis buffer (250 mM NaCl, 50 mM Tris·HCl, pH 8.0, 0.5% Nonidet P-40, 0.2 mM PMSF, 2 *μ*g/ml aprotinin and 2 *μ*g/ml leupeptin, 5 mM NaF and 0.5 mM NaVO_4_), sonicated briefly and then centrifuged at 14 000  × *g* for 15 min at 4 °C. Supernatants were collected and protein concentrations were determined by Bio-Rad protein assay reagent (Bio-Rad, Hercules, CA, USA). For western blotting, samples were separated by SDS-PAGE, transferred to polyvinylidene difluoride membranes (NEN Life Sciences, Waltham, MA, USA), and hybridized to an appropriate primary antibody and horseradish peroxidase-conjugated secondary antibody for detection by enhanced chemiluminescence (Thermo Fisher Scientific Inc., Rockford, IL, USA). For immunoprecipitation, H1299 cells were lysed in 0.25% NP-40 buffer (20 mM Tris-HCl, pH 7.8, 125 mM NaCl, 0.25% Nonidet P-40, 0.2 mM EDTA, 5 mM MgCL2, 1 mM PMSF, 5 mM NaF, 2 mM Na3VO4), 2.0 mg/ml total protein were incubated with anti-FLAG M2 Affinity Gel (A2220, Sigma) for 8 h. For immunoprecipitation in U2-OS cells, 2.0 mg/ml total protein was first precleared with normal mouse or rabbit IgG (Santa Cruz Biotechnology, Santa Cruz, CA, USA) for 1 h, followed by the addition of agarose-conjugated Protein A/G beads (Santa Cruz Biotechnology) and incubated for 30 min with mild agitation. Supernatants were then incubated with a specific primary antibody or a control IgG for 2 h, followed by the addition of agarose-conjugated protein A/G beads (Santa Cruz Biotechnology) and incubated for 1 h. Immune complexes were then washed three times with lysis buffer and resolved by SDS-PAGE and western blotting. All incubations were performed at 4 °C. Antibodies used for western blotting include Pan-Rb (G3-245, BD Biosciences, San Jose, CA, USA; and a mixture of monoclonal antibodies XZ77 and XZ91), hypophosphorylated Rb (G99-549; BD Biosciences), phosphorylated Ser780 (pS780), pS795 or pS807/811 Rb (#9307, #9301 and #9308, respectively; Cell Signaling, Danvers, MA, USA), Pin1 (Ab-1, Oncogene Research Products, Cambridge, MA, USA; H123, sc-15340, Santa Cruz Biotechnology), and Actin (C-11, Santa Cruz Biotechnology). For immunofluorescence, cells were fixed with 4% paraformaldehyde, immunostained with primary Rb antibody and secondary Alexa Fluor 488 (A11001; Santa Cruz Biotechnology) antibodies in 4% BSA, and counterstained with 4,6-diamidino-2-phenylindole (DAPI). For analysis of Rb association with nuclear matrix, cells were treated with or without extraction buffer (250 mM NaCl, 10 mM HEPES-KOH, pH 7.9, 0.1% Triton X-100, 0.5 mM dithiothreitol, 0.2 mM PMSF, 2 *μ*g/ml aprotinin and 2 *μ*g/ml leupeptin, 5 mM NaF and 0.5 mM NaVO_4_) before immunofluorescence. Cell images were recorded with an Axiovert 200 M microscope (Carl Zeiss, Oberkochen, Germany) and analyzed with Axiovision 3.1 software (Carl Zeiss).

### GST pull-down assays

GST pull-down assays were performed essentially as previously described.^58^ In brief, cells were lysed in EBC_200_ buffer (same as EBC_250_, but containing 200 mM NaCl) supplemented with 10% (v/v) glycerol. Equal amounts of proteins were incubated with 20 *μ*l Glutathione-Sepharose 4B Beads loaded with various GST-Pin1 proteins or the control GST for 3 h at 4 °C. Beads were then washed with EBC_200_ buffer and subjected to western blot analyses.

### *In vitro* phosphorylation and dephosphorylation

First, 400 ng of RbC protein (Cell Signaling, #6022) were labeled by *in vitro* phosphorylation in a reaction system containing 40 ng Cyclin E/CDK2 (Cell Signaling, #7524), 30 *μ*Ci *γ*-[^32^P]-ATP (NEN Life Sciences), 100 *μ*M ATP, 50 mM Tris-HCl pH 7.5, 10 mM MgCl_2_, 1 mM EGTA, 2 mM DTT and 0.01% (w/v) Brij-35 by incubation at 30 °C for 30 min. The reaction was stopped by increasing the temperature to 65 °C for 10 min. Free *γ*-[^32^P]-ATP was removed by centrifugation through three Sephadex-G25 columns (Roche) at 3200 r.p.m. for 10 min. Second, to determine the effect of Pin1 on Rb dephosphorylation *in vitro*, 200 ng of *in vitro* phosphorylated RbC protein were incubated with either BSA (0.04 *μ*g/*μ*l), 10 nM okadaic acid, or purified GST-Pin1 protein (0.04 *μ*g/*μ*l) in 50 *μ*l dephosphorylation buffer (10% Glycerol, 20 mM MOPS pH 7.4, 0.1 M NaCl, 1 mM MgCl_2_, 0.1 mM MnCl_2_, 60 mM *β*-mercaptomethanol, 1 mM EGTA, 1 mM DTT and 0.1 *μ*g/*μ*l BSA) at 30 °C for 15 min. Then, 5.0 ng purified PP2A (Upstate Biotechnology, Lake Placid, NY, USA) were added to the reaction mixture, bringing the final volume to 100 *μ*l and incubated at room temperature. Aliquots were removed every 10 min and subsequently resolved by SDS-PAGE. [^32^P]-labeled RbC was detected by autoradiography.

### Flow cytometry

Cells were first incubated in Buffer A (4.0 mM Sodium citrate pH 7.8, 0.1% Triton-X-100, 100 *μ*g/ml RNase A and 50 *μ*g/ml propidium iodide) at room temperature for 10 min. An equal volume of Buffer B (400 mM NaCl, 0.1% Triton-X-100, 50 *μ*g/ml PI) was added and incubated overnight at 4 °C. Cells were then subjected to flow cytometry analysis by FACScan Flow Cytometer (Becton Dickinson Biosciences, Franklin Lakes, NJ, USA), and the data were analyzed using Cell Quest software (Becton Dickinson Biosciences).

### RDS assay

For RDS assays, cells were incubated with methyl-[^14^C]-thymidine (10 nCi/ml) (NEN Life Sciences) for 24 h for labeling. Cells were then synchronized as described above, and subjected to graded doses of *γ*-irradiation. After 45 min, cells were pulse labeled with 2.0 *μ*Ci/ml methyl-[^3^H]-thymidine (NEN Life Sciences) for 15 min. Cells were washed once with ice-cold PBS and three times with 5% trichloroacetic acid and then air dried at room temperature overnight and harvested. Radioactivity was measured in a liquid scintillation counter, and DNA synthesis was represented as the ratio of [^3^H] counts over [^14^C] counts and normalized to the corresponding control.

### BrdU incorporation assay

Cells were labeled with 10 *μ*M BrdU (Roche) for 3 h, fixed with 70% ethanol supplemented with 15 mM glycine pH 2.0 for 30 min at −20 °C, and immunostained with anti-BrdU antibody (Roche) followed by staining with Cy3-conjugated goat anti-mouse IgG (Jackson ImmunoResearch Laboratories, West Grove, PA, USA; 115-165-146) and counterstained with DAPI. BrdU-positive cells were scored under a fluorescent microscope. Three different fields were analyzed, and at least 100 nuclei were counted per field. Results were normalized to untreated controls.

### Assays for senescence-associated phenotypes

For Rb growth suppression assays, Saos-2 cells were co-transfected with pCMV-Rb and/or PLVX-Pin1 (WT or mutant) by Lipofectamine 2000 (Invitrogen Inc.) and selected by puromycin resistance (0.5 *μ*g/ml). Stable cells were grown in normal growth media for 10 days. Cells were then visualized under a light microscope and assessed for percentage of large, flat cell morphology. For senescence-associated *β*-Galactosidase (SA-*β*-Gal) staining, cells were fixed with 3.7% formaldehyde in PBS for 15 min at room temperature, washed and stained with X-Gal solution (Beyotime, Shanghai, China) for 16 h at 37 °C. Cells were then visualized under a light microscope and assessed for percentage of *β*-Gal-positive cells.

### Immunohistochemistry analyses

Paraffin-embedded tissue sections of human breast cancer biopsy samples were purchased from West China Hospital of Sichuan University (Chengdu, Sichuan, China). For immunohistochemistry, slides were dewaxed in xylene, rehydrated in ethanol and then pretreated with 1% H_2_O_2_/10% methanol in PBS for 15 min at room temperature to quench endogenous peroxidase activity. Sections were blocked with 2% BSA for 1 h at room temperature, followed by incubation with anti-Pin1 (H123; Santa Cruz Biotechnology) or anti-pS807/811-Rb (#9308; Cell Signaling) overnight. After extensive washing, the slides were incubated with biotinylated horse anti-rabbit IgG (BA-1000; Vector Laboratories, Burlingame, CA, USA). Reaction products were visualized with the ABC Elite kit (Vector Laboratories) according to manufacturer's instructions. Slides were then counterstained with hematoxylin and eosin (Sigma). Double-blind scoring was then performed for Pin1 and Rb according to intensity of staining and percentage of positive cells. Statistical analysis was performed by Spearman correlation rank test.

## Abbreviations

Rb: Retinoblastoma protein
RbLP: Rb large pocket domain
RbC: Rb C-terminal pocket domain
RbSP: Rb small pocket domain
CDK: Cyclin-dependent kinases
PP1: Protein phosphatase 1
PP2A: Protein phosphatase 2A
PPIase: Peptidyl-prolyl isomerase
pS/T-P: phosphorylated serine/threonine-proline motif
S/T-P: Serine/threonine-proline motif
MEF: Mouse embryonic fibroblast
BSA: Bovine serum albumin
SA-*β*-Gal: Senescence-associated *β*-Galactosidase
St: SV40 small t antigen
RDS: Radioresistant DNA synthesis assays
PBS: Phosphate-buffered saline
