# Supplementary material for: Pin1 inhibits PP2A-mediated Rb dephosphorylation in regulation of cell cycle and S-phase DNA damage
Source: Cell Death Dis. 2015 Feb 12;6(2):e1640–. doi: 10.1038/cddis.2015.3 (PMC4669794; doi:10.1038/cddis.2015.3)
Supplement: Supplementary Information [file cddis20153x1.pdf]

Supplementary Files

Supplementary Figure S1 Consecutive sections from clinical breast cancer samples were subjected to immunohistochemistry for Pin1 expression or hyperphosphorylated Rb (pS807/811) levels. Micrographs were then analyzed and classified as high or low, depending on intensity of staining and percentage of positive cells. Representative images for each cancer case are shown.

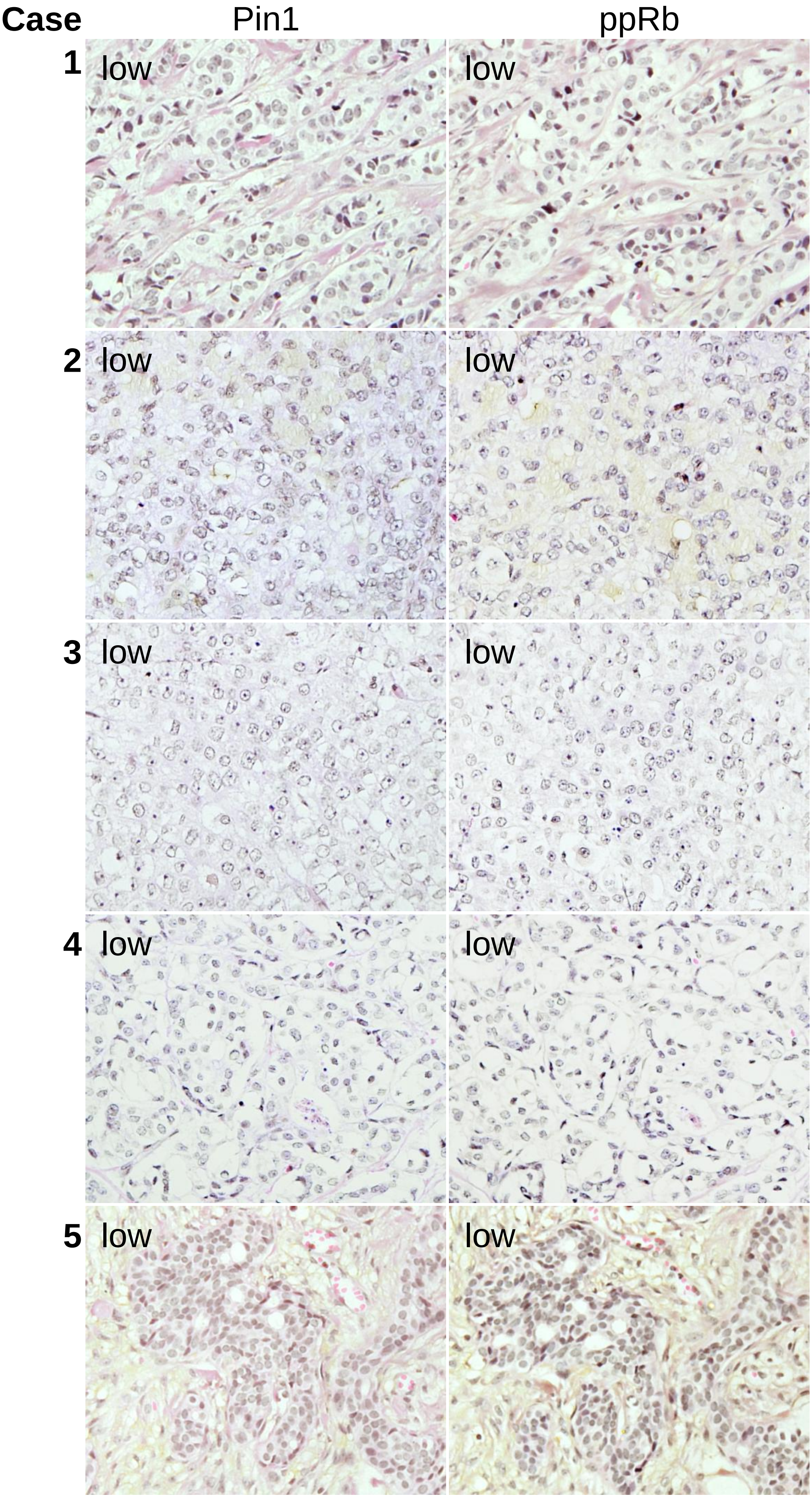
Case
Pin1
ppRb
1
low
low
2
low
low
3
low
low
4
low
low
5
low
low

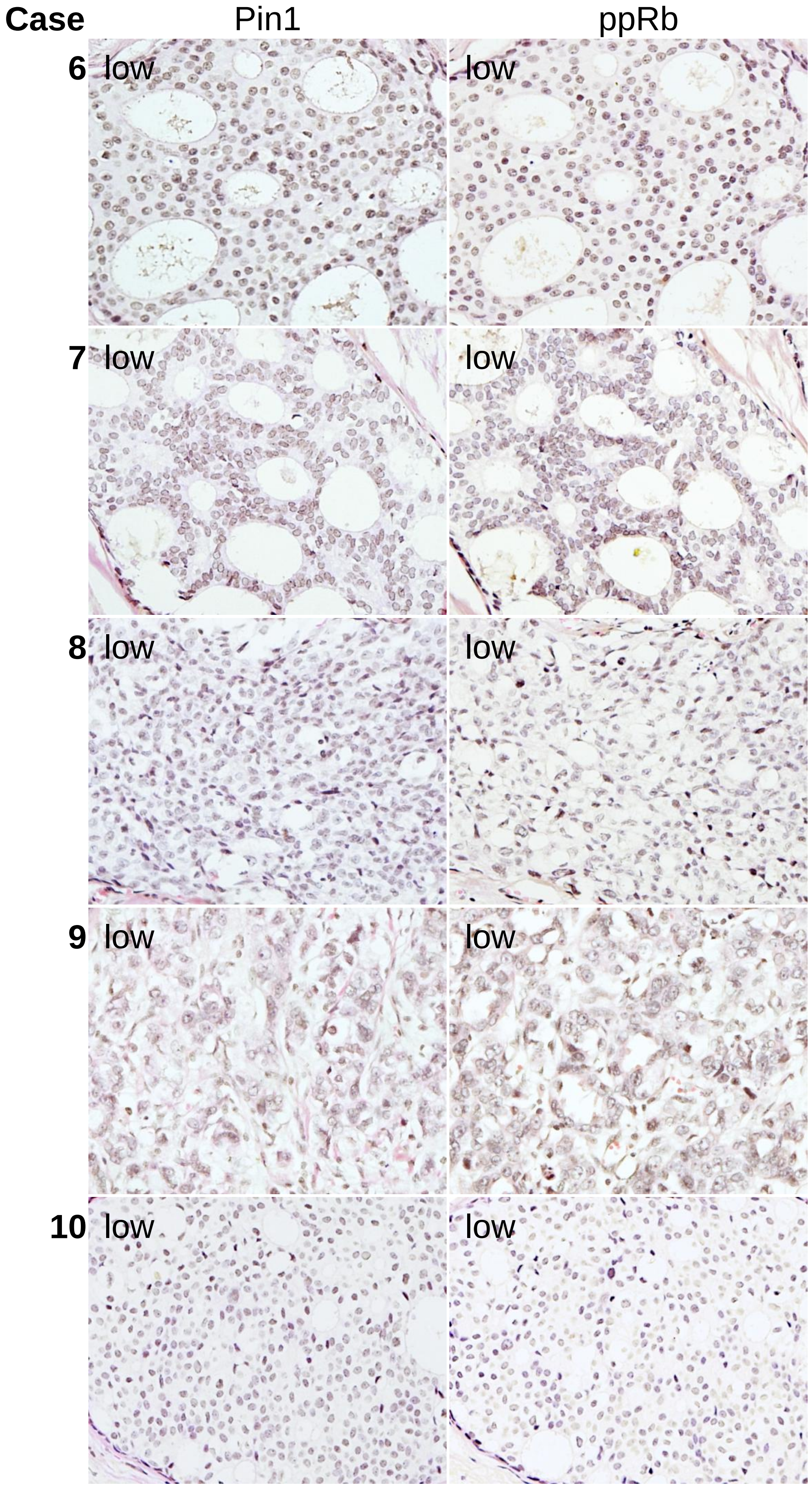

Case
Pin1
ppRb
6
low
low
7
low
low
8
low
low
9
low
low
10
low
low

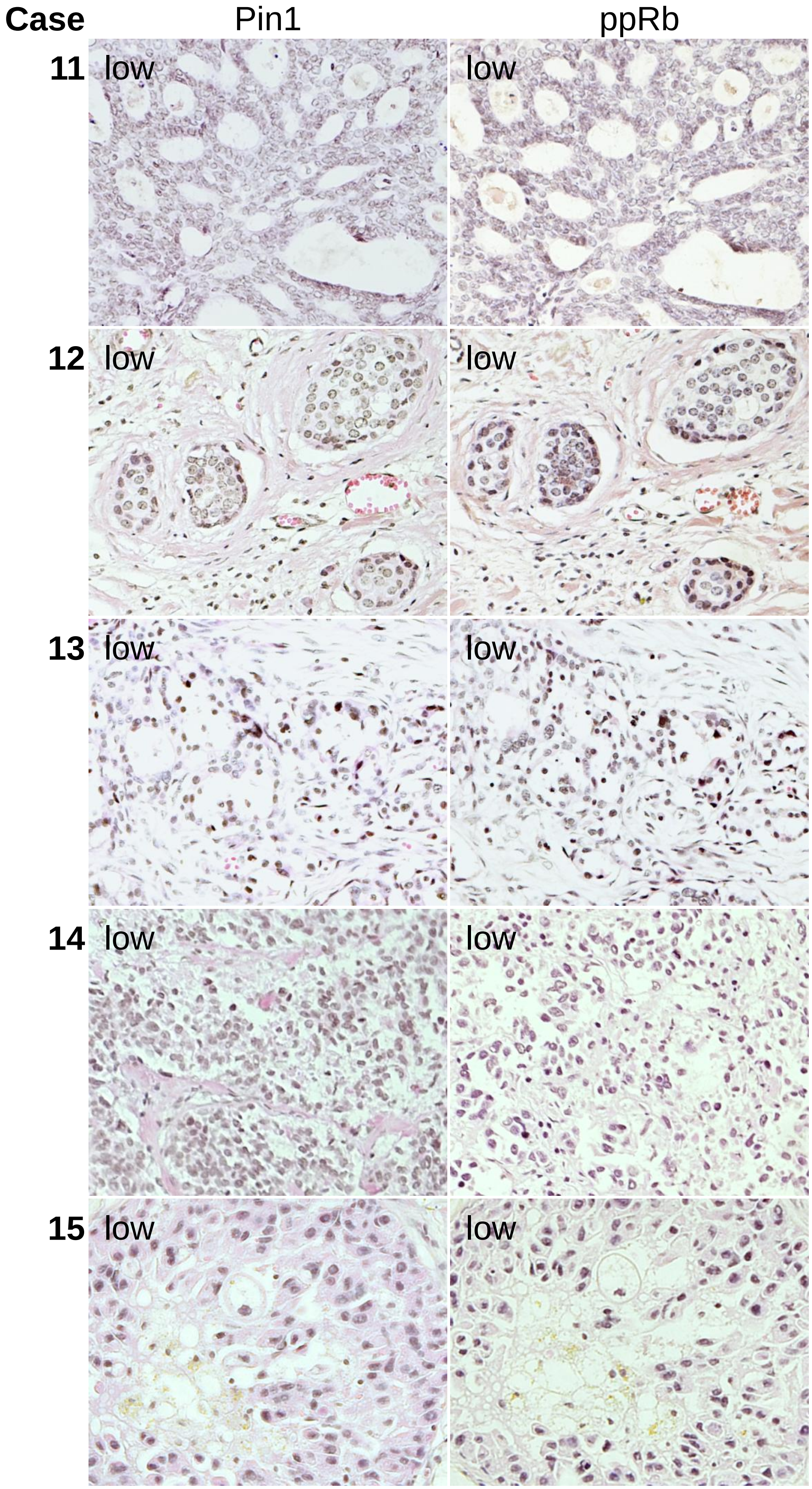
Case
Pin1
ppRb
11
low
low
12
low
low
13
low
low
14
low
low
15
low
low

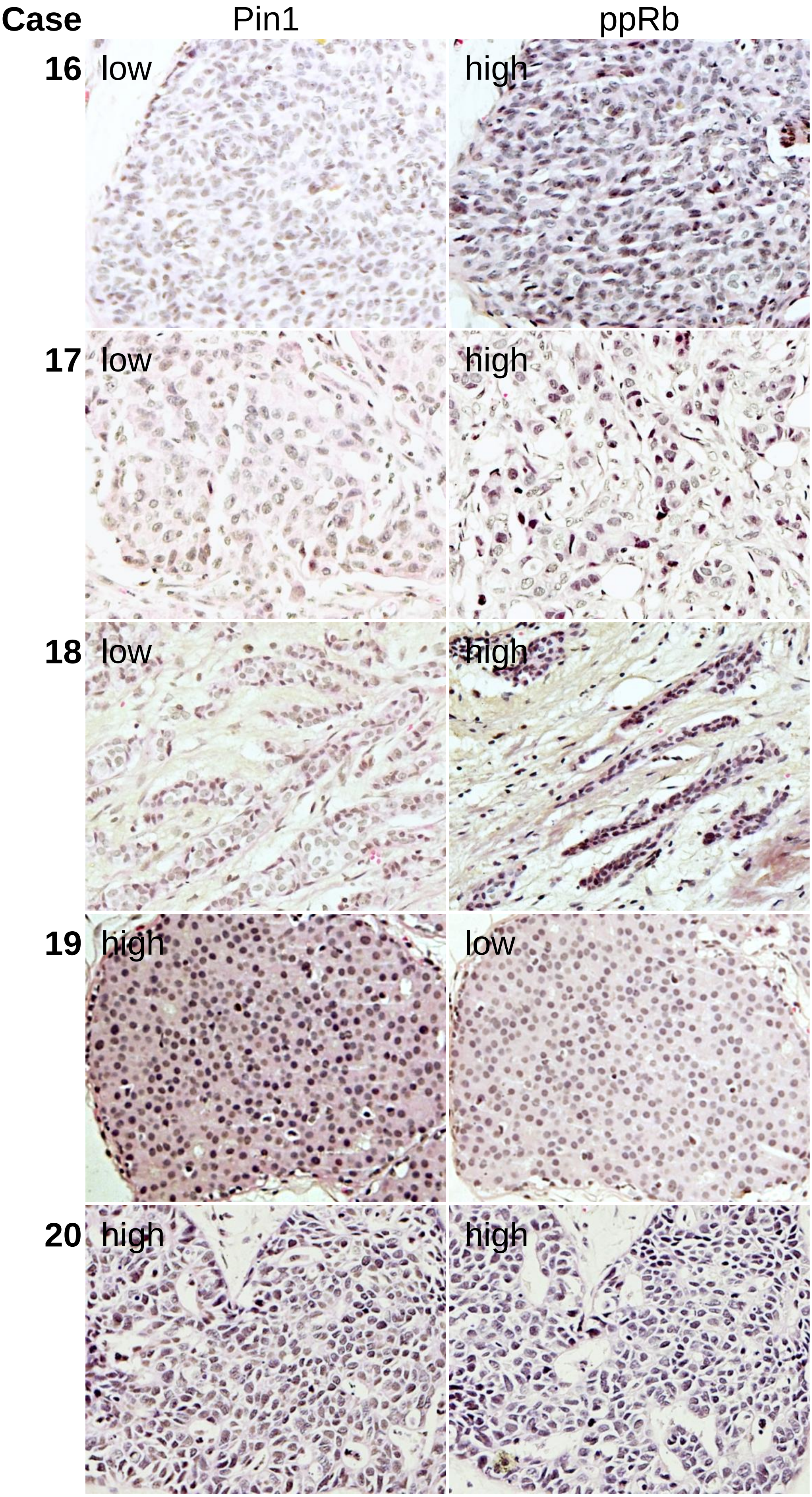
Case
Pin1
ppRb
16
low
high
17
low
high
18
low
high
19
high
low
20
high
high

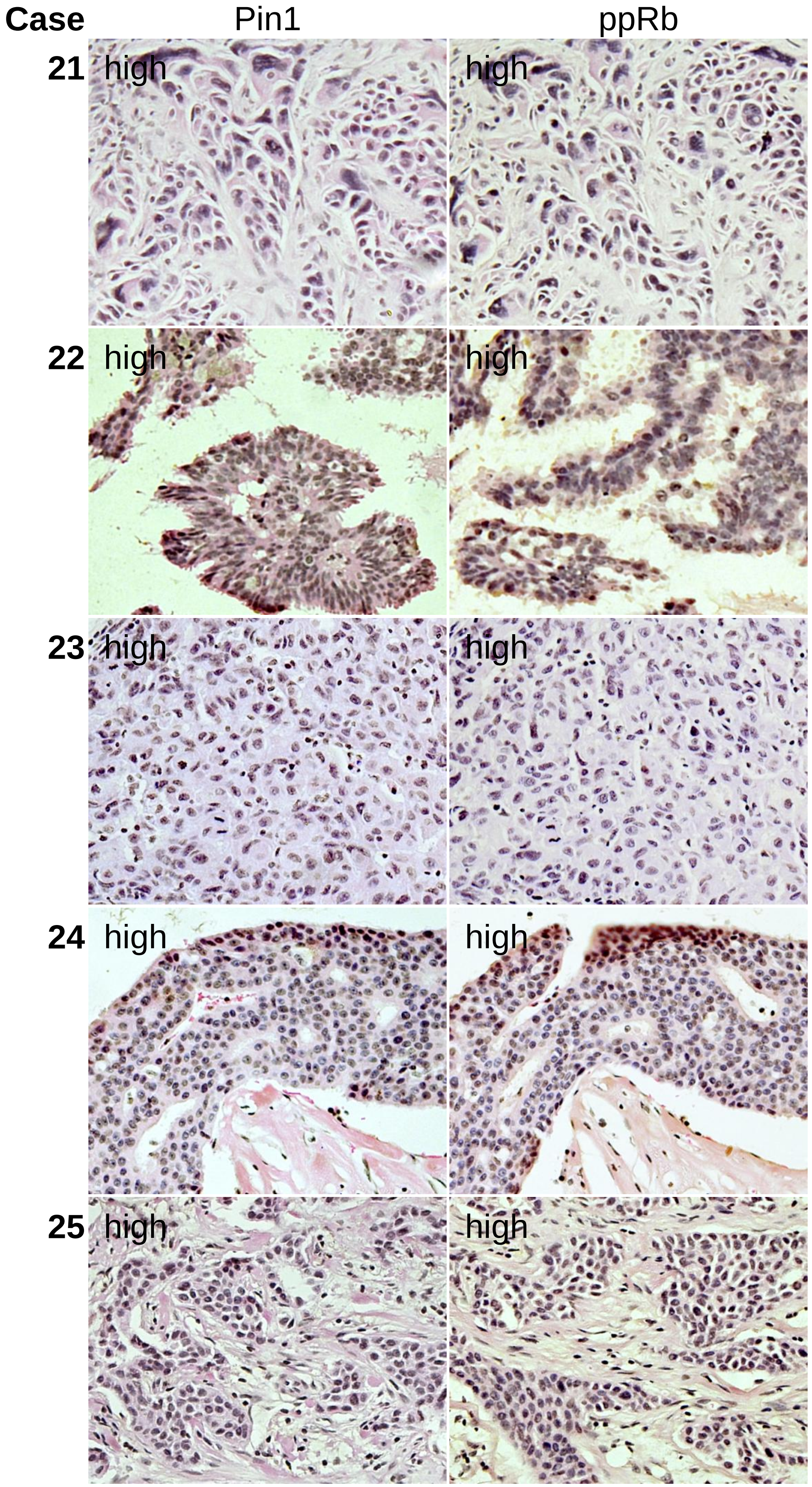
Case
Pin1
ppRb
21
high
high
22
high
high
23
high
high
24
high
high
25
high
high

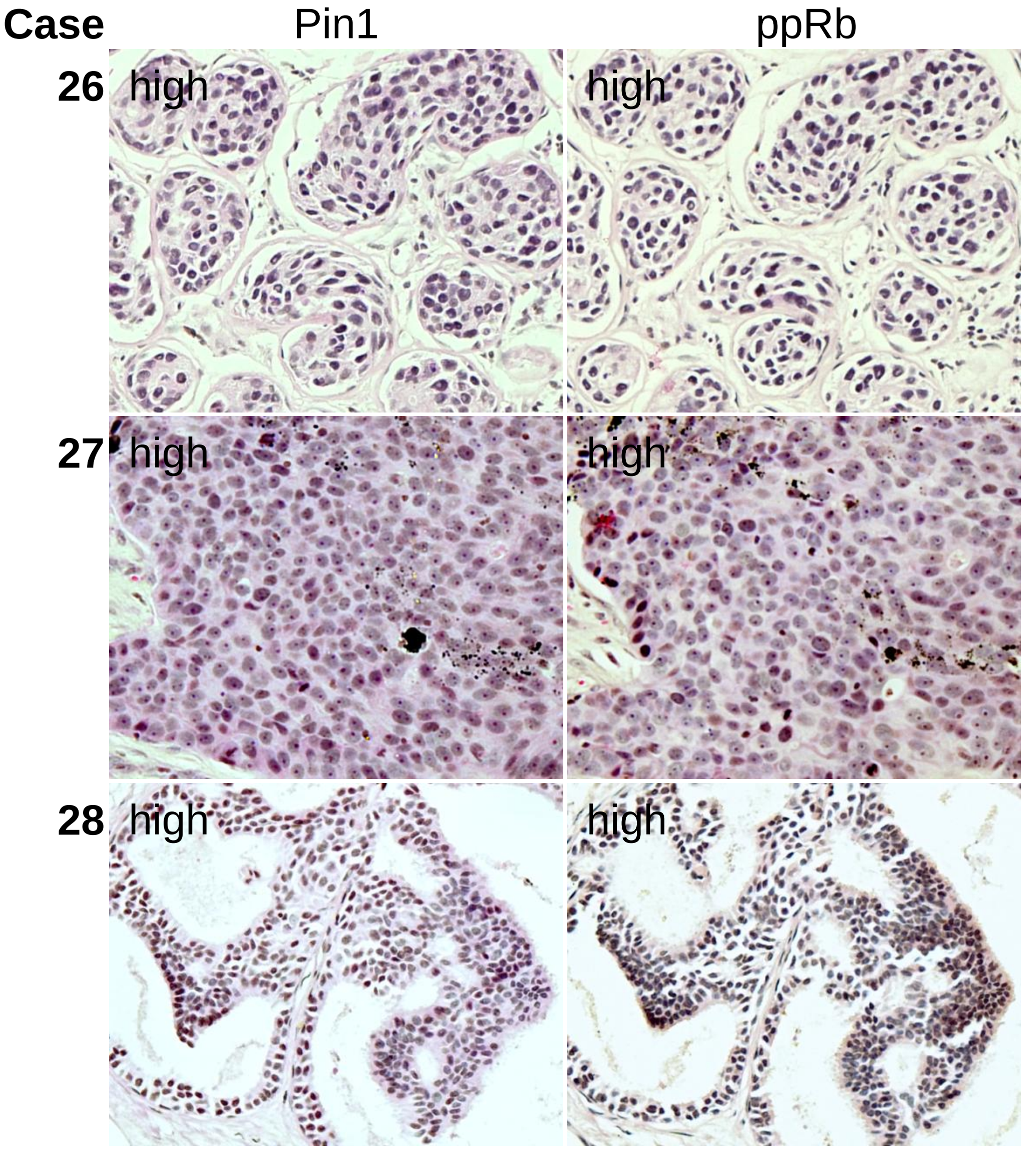
Case
Pin1
ppRb
26
high
high
27
high
high
28
high
high
